# 3*d*-4*f* coupling and multiferroicity in frustrated Cairo Pentagonal oxide DyMn_2_O_5_

**DOI:** 10.1038/s41598-017-15150-w

**Published:** 2017-11-06

**Authors:** S. Chattopadhyay, S. Petit, E. Ressouche, S. Raymond, V. Balédent, G. Yahia, W. Peng, J. Robert, M.-B. Lepetit, M. Greenblatt, P. Foury-Leylekian

**Affiliations:** 1 INAC-MEM, CEA-Grenoble and Université Grenoble Alpes, F-38000 Grenoble, France; 2 Laboratoire Léon Brillouin, CEA, CNRS, Université Paris-Saclay, CE-Saclay, F-91191 Gif-sur-Yvette, Cedex France; 3 Laboratoire de Physique des Solides, CNRS, Université Paris-Sud, Université Paris-Saclay, 91405 Orsay, cedex France; 4 Institut Néel, CNRS and Université Grenoble Alpes, 38042 Grenoble, cedex 9 France; 5 Department of Chemistry and Chemical Biology, Rutgers, the State University of New Jersey, Piscataway, NJ 08854 USA

## Abstract

In solid state science, multifunctional materials and especially multiferroics have attracted a great deal of attention, as they open the possibility for next generation spintronic and data storage devices. Interestingly, while many of them host coexisting 3d and 4f elements, the role of the coupling between these two magnetic entities has remained elusive. By means of single crystal neutron diffraction and inelastic neutron scattering experiments we shed light on this issue in the particular case of the multiferroic oxide DyMn_2_O_5_. This compound undergoes a first order magnetic transition from a high temperature incommensurate phase to a low temperature commensurate one. Our investigation reveals that although these two phases have very different magnetic structures, the spin excitations are quite similar indicating a fragile low temperature ground state with respect to the high temperature one. Such a rare scenario is argued to be a manifestation of the competition between the exchange interaction and 4*f* magnetic anisotropy present in the system. It is concluded that the magnetic structure, hence the ferroelectricity, can be finely tuned depending on the anisotropy of the rare earth.

## Introduction

Magnetic ferroelectrics, where the onset of ferroelectricity essentially requires the spins to be ordered, are a fascinating class of materials^1^. The associated physics is undoubtedly challenging because of the coupled spin, charge, and lattice degrees of freedom. Particularly, the issue of determining the actual role of spin in the emergence of ferroelectric state has been a very crucial topic of research in recent years. Different mechanisms have been proposed, like the *inverse Dzyaloshinskii*-*Moriya* (*DM*) *interaction* model^2^, the *exchange*-*striction* mechanism^1^ and the *spin dependent metal*-*ligand hybridization* model^3–6^ among others, but no universal model could be implemented to the entire family. It remains that the magnetic properties of many of such magnetic ferroelectrics are driven by 4*f* rare earths and 3*d* transition metal ions. There is thus some urgency in understanding the role of the 3*d*-4*f* coupling. Noting the fact that 4*f* elements are also well known for their exotic electric properties, for their usage in various laser based applications and also as permanent magnets (NdFeB and SmCo for instance), understanding the coupling between the two magnetic species would significantly benefit the wing of material engineering as far as the technological implementation is concerned.

In this report, we have chosen to study DyMn_2_O_5_, a spin induced multiferroic system of the RMn_2_O_5_ (R = Y, Rare earths) family showing large spontaneous electric polarization and colossal magnetoelectric effect, one of the strongests in the entire family^1,7–14^. With the coexistence of 4*f* and 3*d* metals, this composition appears to be an ideal seedbed to investigate the 3*d*-4*f* coupling in a very prominent way.

DyMn_2_O_5_ is mainly characterized by two transitions. Below *T*_1_ = 42 K, it undergoes a magnetic phase (HT phase) transition with a propagation vector *k*_1_ = (1/2 − *δ*(*T*), 0, 1/4 − *ε*(*T*))^12,15^. Ferroelectric order arises below 40 K which is very close to the HT transition. With lowering temperature, a first order phase transition occurs at *T*_2_ = 8 K, from the HT phase to a low temperature commensurate magnetic phase (LT) with *k*_2_ = (1/2, 0, 0). This transition is believed to be associated with the ordering of Dy^3+^ moments. In few literatures, the existence of a *lock in* transition around 18 K was also reported^12^. Although very weak, the HT phase persists below *T*_2_ and coexists with the LT phase. In spite of a few previous attempts, revealing the magnetic structures of different phases has remained a dubious issue for this compound^12,15–17^. In this study, we conclusively determine the magnetic structure of both the HT and LT phases, by means of detail single crystal neutron diffraction experiments. In addition, inelastic neutron scattering investigations were carried out to characterize the spin dynamics. It is found that the spin excitations of the HT and LT phases are surprisingly very similar, showing especially a soft mode along (3/2, 0, $\ell$).

**Figure 1:**
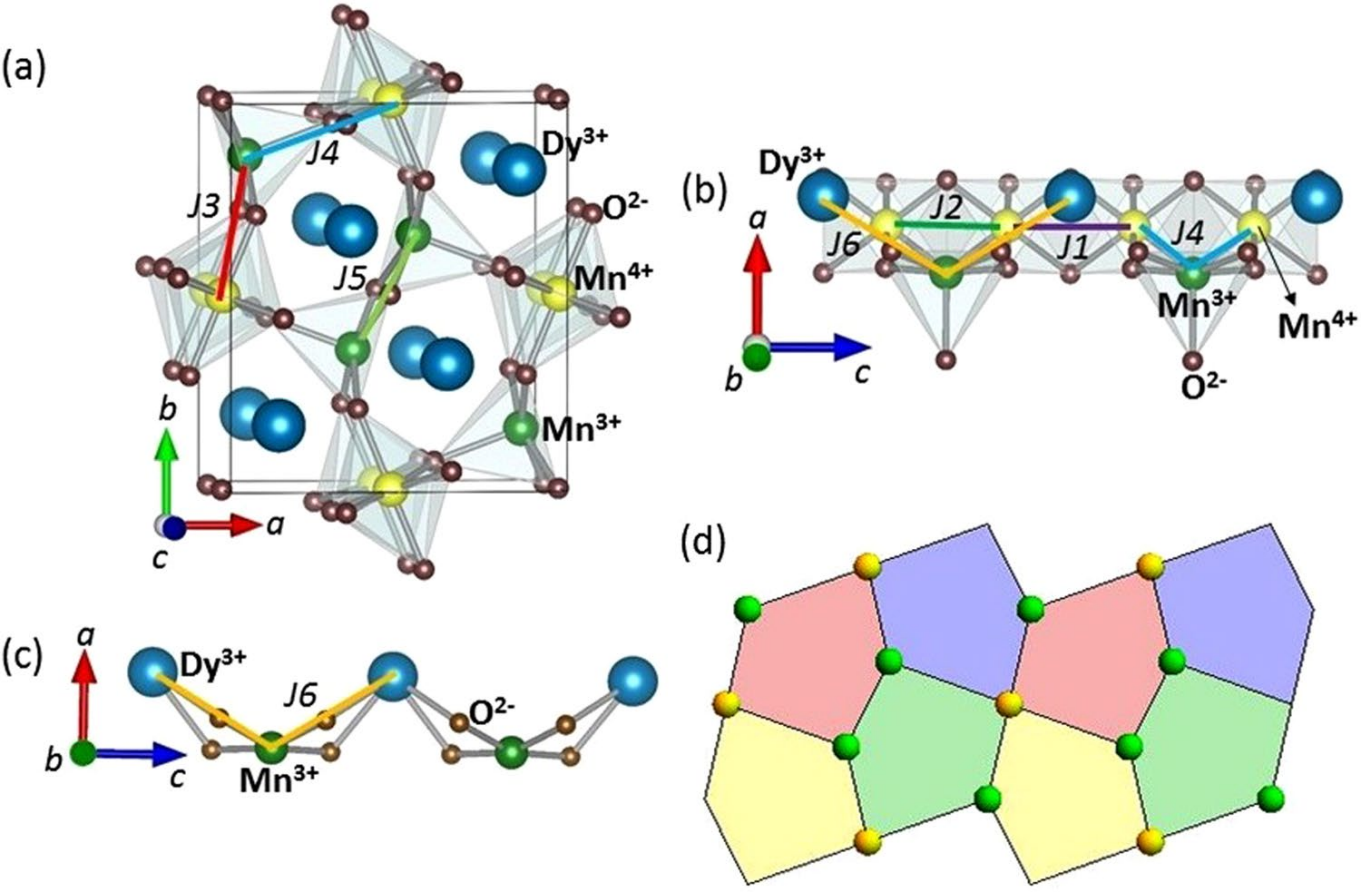
(**a**) A perspective view of the crystallographic structure and the Mn-Mn magnetic exchange interactions in the (*a*, *b*) plane for DyMn_2_O_5_. (**b**) The Mn^4+^ chain along *c* and the dominant Mn-Mn exchange interactions in the (*a*, *c*) plane. It also shows Mn^3+^-Dy^3+^ interaction *J*_6_ along *c*. (**c**) Detailed path of interaction for *J*_6_. (**d**) Shows the Cairo pentagonal tiling in the (*a*, *b*) plane.

As claimed in a recent study, DyMn_2_O_5_ belongs to ferroelectric Pm group^18^ but only differs from the *Pbam* orthorhombic space group by weak deformations. DyMn_2_O_5_ is formed by Mn^3+^O_5_ square pyramids and corner sharing Mn^4+^O_6_ octahedra^8,9^. In the (*a*, *b*) plane, loops of five Mn ions (three Mn^3+^ and two Mn^4+^) are formed with three inequivalent nearest neighbor antiferromagnetic (AFM) interactions: *J*_3_, and *J*_4_ couple the Mn^3+^ and Mn^4+^ ions while *J*_5_ couples the Mn^3+^ (see Fig. 1a). In contrast, along *c*, ribbons of Mn^4+^ are formed in presence of *J*_1_ (via Dy^3+^ layers) and *J*_2_ (via Mn^3+^ layers) interactions, as shown in Fig. 1b. Interestingly, the magnetic frustration inherent to all multiferroic materials is here due to the “Cairo pentagonal lattice” formed by Mn^3+^ and Mn^4+^ ions (see Fig. 1d and ref.^19^). Such lattices have become famous in the problem of tiling a plane with regular polygons, but also after the beautiful streets in the city of Cairo paved in this design.

## Results

### Solving the magnetic structures: LT and HT phases

**Figure 2:**
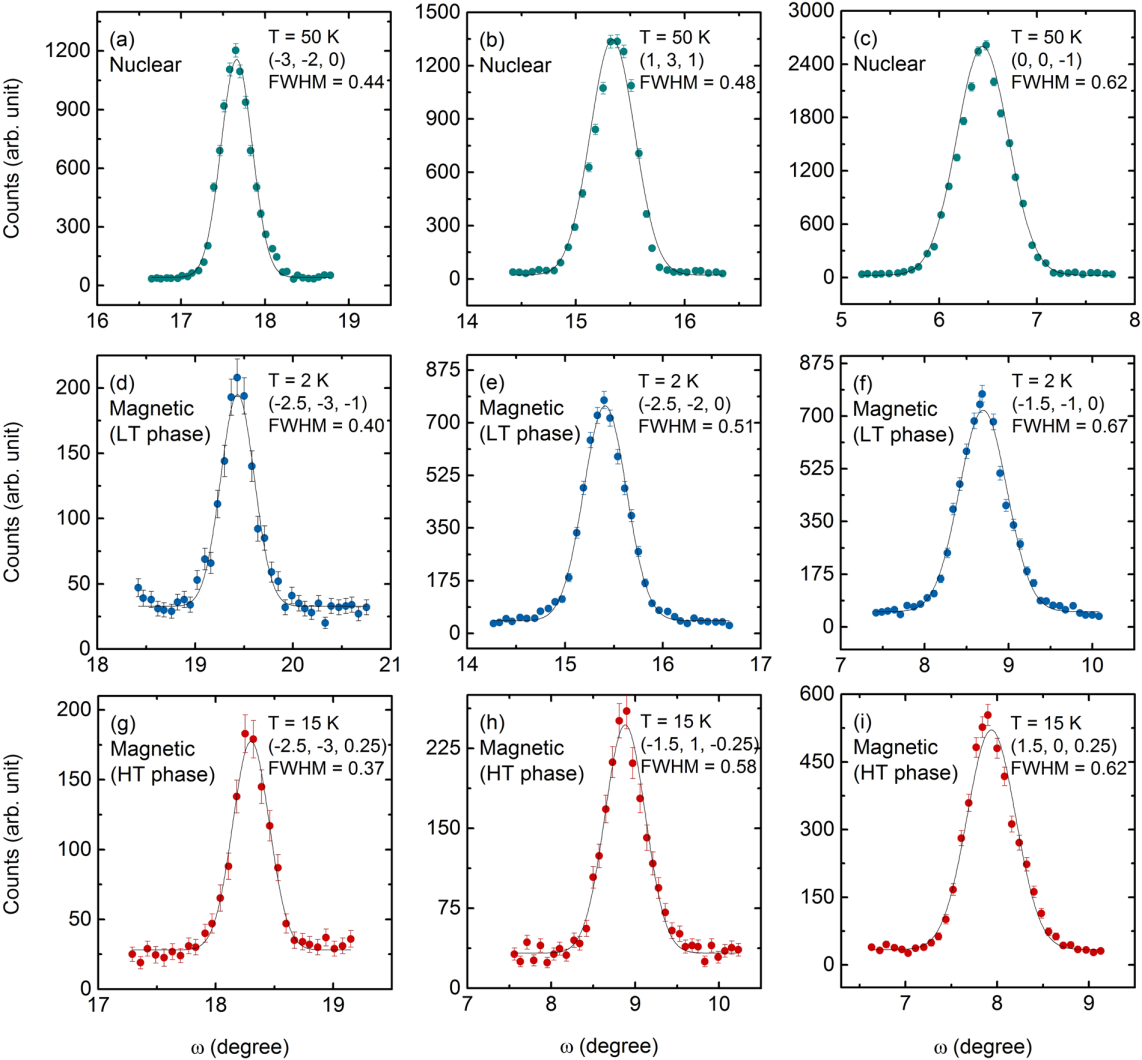
Some of the representative rocking curves from the single crystal neutron diffraction experiment. (**a**–**c**) Nuclear reflections measured at 50 K, (**d**–**f**) magnetic reflections corresponding to the LT phase measured at 2 K, and (**g**–**i**) magnetic reflections corresponding to the HT phase measured at 15 K. Solid lines are fit to the curves with a Gaussian function. The estimated full width at half maximum (FWHM) is also shown for each measured reflection.

We performed single crystal neutron diffraction operated in four circles geometry using a crystal with size nearly 2.5 × 1.7 × 1 mm^3^. About 400 to 500 nuclear and/or magnetic Bragg reflection’s integrated intensities were estimated at 50 K, 25 K, 15 K, and 2 K by measuring rocking curves around each reflections. Figure 2 shows a few representative rocking curves fitted with Gaussian function. The curves presented could be categorized into three distinct groups: (a)–(c) nuclear reflections, (d)–(f) LT phase magnetic reflections, and (g)–(i) HT phase magnetic reflections. From the fit it is evident that the nuclear and magnetic reflections with nearly equal *ω* values have practically identical peak widths as expected for long range magnetic order. The refinement of the angular positions of the 50 K (above the magnetic transition) nuclear reflections revealed that the lattice constants are quite close to the one reported at room temperature. These values also match well with the literature reported earlier using powder sample^15^. Refinements of the nuclear and magnetic structures at 2 K and 15 K were performed using the FullProf software package^20^. We used the formalism of Becker-Coppens to refine the extinction in the crystal. The pretty low values of the discrepancy factors ensure the quality of the refinement we achieved. The scale factors obtained from the refinements were used in the refinements of the magnetic structures at 15 K and 2 K.

**Figure 3:**
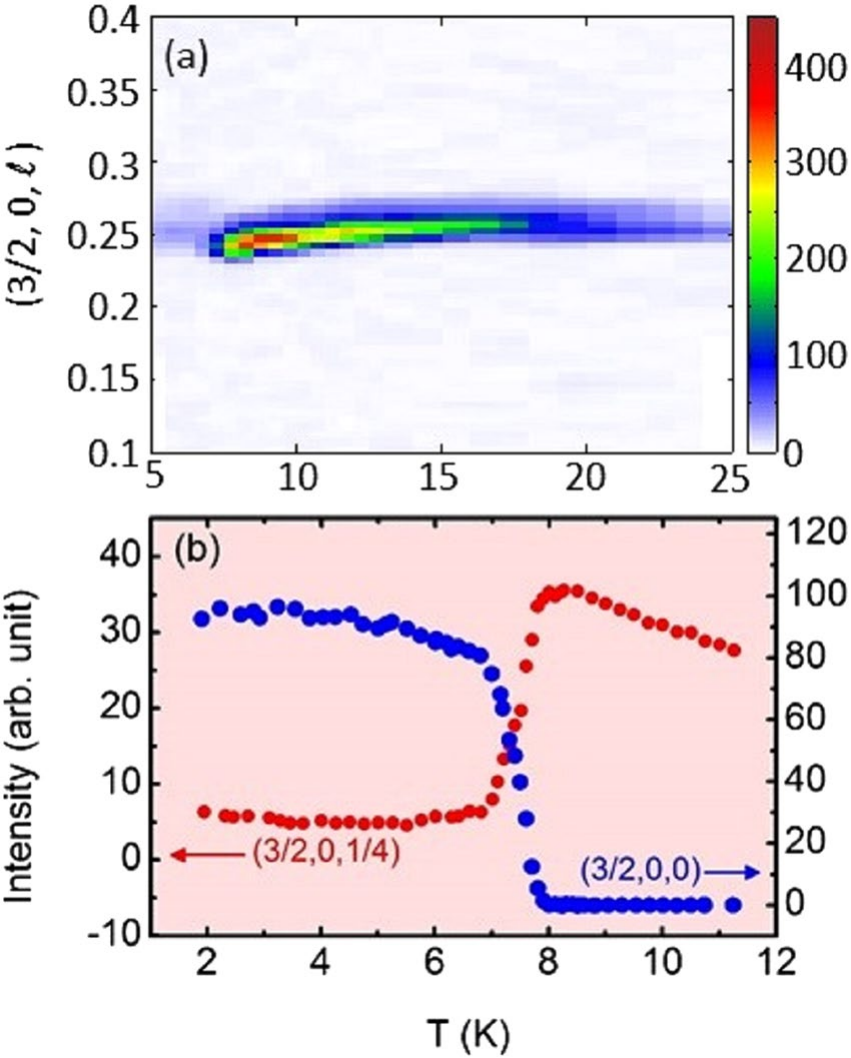
(**a**) A color map constructed by combining the *Q*-scans along $(1.5,0,\ell )$ with varied temperature. Thermal dependence of integrated intensities for two magnetic satellites (**b**) (3/2, 0, 1/4) and (3/2, 0, 0) across the transition at 8 K.

To follow the temperature evolution of the magnetic propagation vectors, *Q*-scans were performed below 45 K. The satellites are only visible below 30 K due to a large background. Two magnetic phases are observed, in agreement with previous reports: above 8 K, an incommensurate phase (HT) with *k*_1_ = (1/2 − *δ*(*T*), 0, 1/4 − *ε*(*T*)) with *δ*(*T*) and *ε*(*T*) being very small and, below 8 K, a commensurate phase (LT) with propagation vector *k*_2_ = (1/2, 0, 0). Figure 3(a) shows *Q*-scans carried out along $(3/2,0,\ell )$ for a series of temperatures in a color-map fashion. *ε*(*T*) increases slightly up to 8 K where the HT-LT transition occurs. Although very weak, the signal of the HT phase persists down to 2 K (in agreement with previous reports)^15^. Figure 3(b) shows the thermal variation of two specific magnetic Bragg reflections (3/2, 0, 1/4) and (3/2, 0, 0), characteristics of the HT and LT phases respectively. It clearly shows that the (3/2, 0, 1/4) reflection becomes extremely weak in comparison with the (3/2, 0, 0) that rises below 8 K. We thus confirm that the magnetic ground state of this compound consists of two coexisting phases: the dominant LT phase along with the very feeble HT phase.

**Figure 4:**
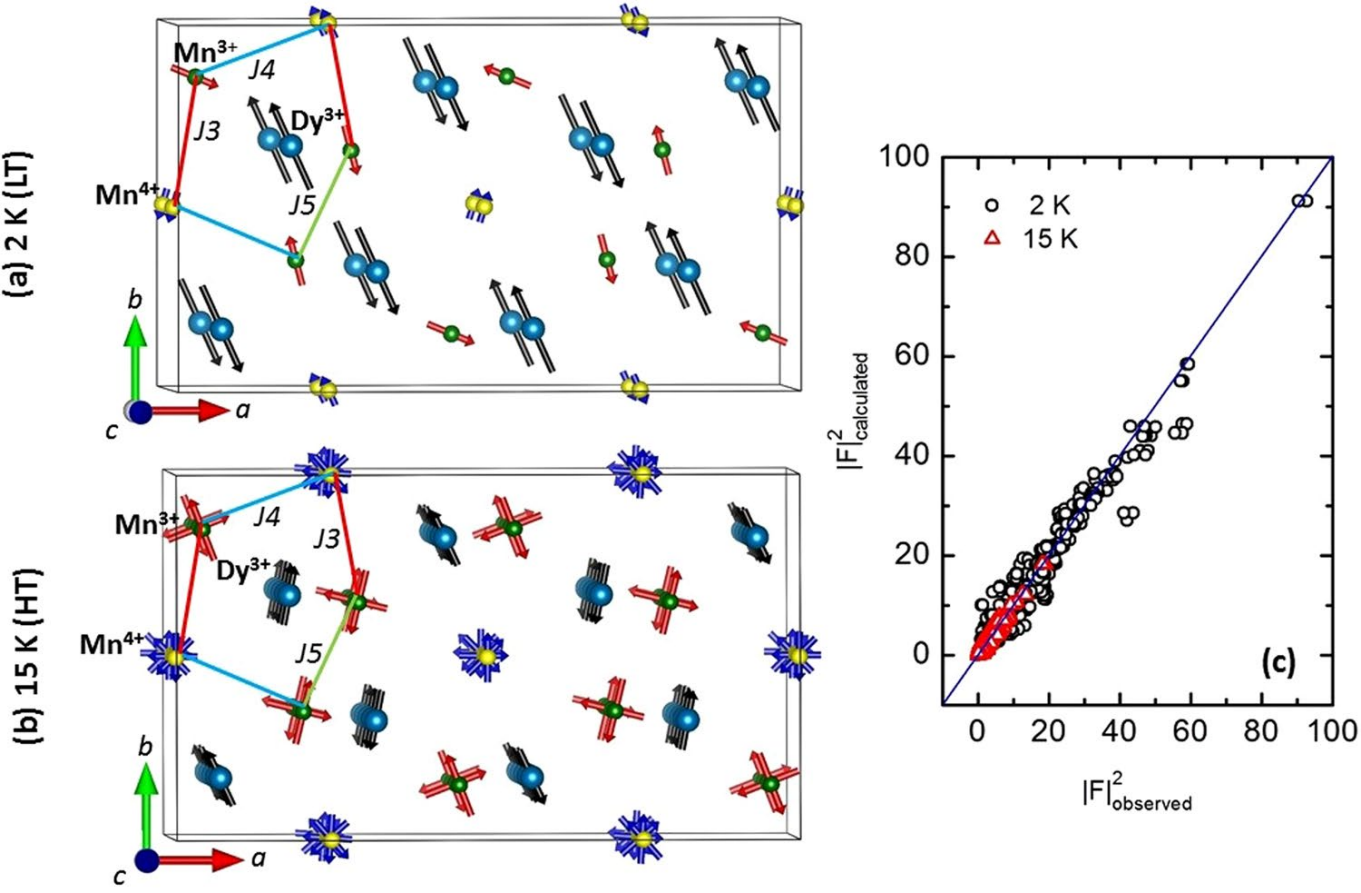
(**a**) Magnetic structure in the LT phase at 2 K where each sublattice is ferromagnetically stacked along *c* direction. (**b**) Magnetic structure of the HT phase refined at 15 K showing the spiral arrangement of Mn^3+^ and Mn^4+^ spins along *c*. Spins of Dy^3+^ show up-up-down-down amplitude modulated structure along *c*. Magnetic interactions *J*_3_, *J*_4_, and *J*_5_ are also shown. (**c**) ${F}_{observed}^{2}$ vs. ${F}_{calculated}^{2}$ plot for both temperatures to depict the quality of refinements.

**Figure 5:**
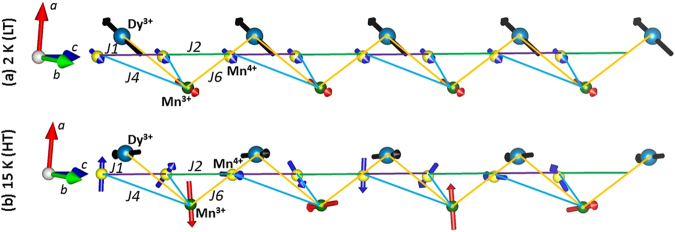
Stacking of spins along *c* direction: (**a**) At 2 K (LT phase), all kinds of spins are parallel. (**b**) At 15 K (HT phase), spins of Mn^4+^ and Mn^3+^ show spiral arrangement. Mn^4+^ spins rotate by 45° from each site to another along *c* whereas it is by 90° in case of Mn^3+^ . In contrary to the Mn spins, Dy^3+^ spins aquire an amplitude modulated structure with ↑↑↓↓ fashion. In addition to the spin stacking, exchange interactions *J*_2_, *J*_4_, and *J*_6_ are also shown.

At 2 K, 500 magnetic Bragg reflections were collected associated with the propagation wave vector *k*_2_ = (1/2, 0, 0). Unfortunately, representation analysis was not of any use to find symmetry relations among moments and thus determine a model of the magnetic structure. Refinement of the LT phase turned out to be challenging in presence of ten uncorrelated magnetic sites corresponding to three magnetic sub-lattices (Dy^3+^ Mn^3+^ and Mn^4+^). It was found that using a *toy model* with magnetic symmetry *P*_2*a*_ *b*′2_1_ *m*′ helped to reduce the free parameters in some of the RMn_2_O_5_ systems with (1/2, 0, *k*_*z*_) propagation vector^15,21^. This magnetic space group is a derivative of the crystallographic group *Pb*2_1_ *m* which was proposed to be the space group in the emerged ferroelectric phase of RMn_2_O_5_ at low temperature^8^. We started the refinement using this model. To make the scenario simpler, we also incorporated few constraints based on literature available for RMn_2_O_5_^15,21^. These constraints included working with a spin density wave (SDW) model, putting all the spins in the (*a*, *b*) plain. In addition, moments of similar type of ions were set to be equal. Testing with different possible orientations among the spins under the constraint relations mentioned above eventually steered us to the solution of the magnetic structure depicted in Fig. 4(a) and in Table 1. A very good agreement factor (*R*_*F*_ = 8.6%) ensures that the proposed model is reliable. The spins are found predominantly along the *b* direction, and arranged in a ferromagnetic fashion along the *c* axis (see also the focus in Fig. 5(a). The non collinearity within the (*a*, *b*) plane is the manifestation of the different frustrated interactions. The obtained structure is close to the one reported by Blake *et al*. using powder neutron diffraction data^15^.

**Table 1:** Refinement result for the magnetic structure of DyMn_2_O_5_ in the LT phase at 2 K with *k*_2_ = (1/2, 0, 0).

*Atom*	Position	*M*_*R*_ (*μ*_*B*_)	*ϕ*(°)	*θ*(°)	Phase (2*π*)
Mn^3+^	(0.4112, 0.3507, 0.5)	2.97(11)	105(3)	90	0
	(0.5888, 0.6493, 0.5)	2.97(11)	285(3)	90	0
	(0.0888, 0.8507, 0.5)	2.97(11)	−22(4)	90	0
	(0.9112, 0.1493, 0.5)	2.97(11)	−22(4)	90	0
Mn^4+^	(0, 0.5, 0.2536)	1.88(5)	253(5)	90	0
	(0, 0.5, 0.7465)	1.88(5)	253(5)	90	0
	(0.5, 0, 0.2536)	1.88(5)	115(1)	90	0
	(0.5, 0, 0.7465)	1.88(5)	115(1)	90	0
*Dy* ^3+^	(0.1387, 0.1719, 0)	7.47(3)	295(1)	90	0
	(0.3613, 0.6719, 0)	7.47(3)	115(1)	90	0
	(0.6387, 0.3281, 0)	7.47(3)	295(1)	90	0
	(0.8613, 0.8281, 0)	7.47(3)	295(1)	90	0

The moments are described using spherical coordinates system with *M*_*R*_, *θ*_*R*_, and *ϕ*_*R*_ being the real Fourier components at the (*x*, *y*, *z*) positions, polar angle, and azimuthal angle respectively. The achieved agreement factor is *R*_*F*_ = 8.6%.

Similar to the situation observed in the LT phase, irreducible representation analysis with propagation vector *q* = (0.5, 0, 0.25) was not useful due to the lack of correlations among the moments. Attempting the LT phase structure in the 15 K (HT phase) data was unsuccessful. The model proposed by Wilkinson *et al*. corresponding to *k*_2_ reflections at 4 K did not appear to be suitable as well^16^. Unlike the situation at 2 K, usage of SDW configuration under Shubnikov group *P*_2*a*_ *b*′2_1_ *m*′ was a failure too, despite its flexibility. However, execution of simulated annealing (SA) analysis helped to have some ideas about the nature of possible couplings with an indication of spirality in the Mn sublattices. Based on that, tests with numerous numbers of possible configurations were made. Such an attempt revealed that incorporating spiral structure descriptions for Mn^3+^ and Mn^4+^ sub-lattices along *c* direction indeed improves the data fitting quality significantly. This spiral arrangement is described by the classical Fourier decomposition: $\vec{S}=\frac{1}{2}(\vec{R}+i\vec{I}).{e}^{-2\pi {\rm{.}}i\psi }$ where $\vec{R}$ and $\vec{I}$ are real and imaginary components respectively. *ψ* is a relative phase factor applicable to each site. We have used spherical polar coordinate system to represent the components of $\vec{R}$ and $\vec{I}$ (hence represented as (*M*_*R*_, *θ*_*R*_, *ϕ*_*R*_) and (*M*_*I*_, *θ*_*I*_, *ϕ*_*I*_) where *M*, *θ*, *ϕ* denote the amplitude, polar angle, and azimuthal angle respectively).

However, usage of a spiral model for Dy^3+^ sublattice deteriorates the quality of fitting. Instead, inclusion of Dy^3+^ spins following SDW approach helps to improve the refinement quality further. It is worth noting that such states where the amplitude of the rare earth ordered magnetic moment is modulated, is a common feature in the RMn_2_O_5_ family^7^. The refined structure at 15 K is shown in Fig. 4(b) and the values of the refined parameters are given in Table 2. The higher value of the *R*_*F*_ (15.7%) factor is a manifestation of much weaker reflections at 15 K. However, the ${F}_{observed}^{2}$ vs. ${F}_{calculated}^{2}$ plot strongly advocates for the satisfactory quality of the refinement (Fig. 4(c)). Two magnetic domains, respectively generated by the (*x*, *y*, *z*) and (−*x*, −*y*, *z*) symmetry operations, have been taken into account, and their volume fraction was refined to 52/48%. Note that *δ* and *ε* are neglected in this approach. The obtained structure differs from two of the possible models made by Johnstone *et al*. using resonant x-ray technique at 15 K, since there is no spiral description in their proposition^17^. It is also different from the model proposed by Ratcliff *et al*. at 22 K where only the Mn^4+^ spins show a spiral arrangement^12^. It is important to mention that attempts of using these previously proposed models were unsuccessful.

**Table 2:** Refinement results for the magnetic structure of DyMn_2_O_5_ in the HT phase at 15 K with *k*_1_ = (0.49, 0, 0.254).

*Atom*	Position	*M*_*R*_ (*μ*_*B*_)	*ϕ*_*R*_°	*θ*_*R*_°	*M*_*I*_ (*μ*_*B*_)	*ϕ*_*I*_°	*θ*_*I*_°	Phase (2*π*)
Mn^3+^	(0.4112, 0.3507, 0.5)	3.07(3)	−194(2)	90	3.07(3)	−104(2)	90	0
	(0.5888, 0.6493, 0.5)	3.07(3)	−14(2)	90	3.07(3)	−284(2)	90	0
	(0.0888, 0.8507, 0.5)	3.07(3)	−159(2)	90	3.07(3)	−69(2)	90	0
	(0.9112, 0.1493, 0.5)	3.07(3)	−159(2)	90	3.07(3)	−69(2)	90	0
Mn^4+^	(0, 0.5, 0.2536)	2.33(2)	−45(2)	90	2.33(2)	−315(2)	90	0
	(0, 0.5, 0.7465)	2.33(2)	0(2)	90	2.33(2)	−270(2)	90	0
	(0.5, 0, 0.2536)	2.33(2)	−4(2)	90	2.33(2)	−274(2)	90	0
	(0.5, 0, 0.7465)	2.33(2)	−319(2)	90	2.33(2)	−229(2)	90	0
*Dy* ^3+^	(0.1387, 0.1719, 0)	3.99(2)	115(1)	90	—	—	—	0.125
	(0.3613, 0.6719, 0)	3.99(2)	79(1)	90	—	—	—	0.125
	(0.6387, 0.3281, 0)	3.99(2)	259(1)	90	—	—	—	0.125
	(0.8613, 0.8281, 0)	3.99(2)	115(1)	90	—	—	—	0.125

The spins have been described with their spherical coordinates (*θ* and *ϕ*). The spiral model encompasses a real (*M*_*R*_) and an imaginary (*M*_*I*_) Fourier component. The agreement factor is *R*_*F*_ = 15.7%.

#### Origin of the HT-LT transition: Numerical mean field calculation

With these results in hand, we now propose a scenario to account for this sequence of magnetic structures. The main idea is that the Dy^3+^ spins are characterized by a strong easy-axis anisotropy that tends to align them along the *b* direction.

There are indeed some salient features which make the magnetic structure at 15 K rather different from the 2 K structure. At 2 K, all the spins are arranged ferromagnetically along the *c* direction, whereas, at 15 K, Mn^4+^ spins rotate by 45° from site to site around the *c* axis, and by 90° in the case of Mn^3+^ ions. Interestingly, Dy^3+^ spins do not prefer to be arranged in a spiral manner. The refined structure at 15 K corresponds to ↑↑↓↓ stacking along *c* of Dy^3+^ spins pointing along the *b* direction. This is a strong argument in favor of a significant anisotropy along that particular axis. It is worth noting that thermal variation of magnetization measurement performed along the three crystallographic directions also indicates a slightly higher magnetization along *b* (not shown). To figure out quantitatively the nature of the Dy^3+^ anisotropy, numerical *ab*-*initio* calculations (including 4*f* electronic correlation and spin-orbit interaction) have been performed, on an embedded DyO_5_ fragment. We used the CASSCF code of the MOLCAS package^22^ for the orbital and correlation calculations, the EPCISO code^23^ for the spin-orbit calculation and a home made code for the anisotropy and magnetic moments calculation from the *ab*-*initio* results. We find that the low energy crystal field states of the Dy^3+^ ions are formed by a doublet. The corresponding |↑〉 and |↓〉 states have an Ising like character and carry a moment *μ* = 〈↑|*τ*|↑〉 = −〈↓|*τ*|↓〉 = 8.5 *μ*_B_ which forms an angle *ϕ* = 77° away from the *a* axis (hence close to the *b* axis). This compares very well with the experimental values of 7.5 *μ*_B_ and 115°. The |↑〉 and |↓〉 states are also well separated from the next excited states, located at 16 meV.

Meanwhile, a closer look at the 2 K structure shows that Mn^3+^ and Dy^3+^ spins are anti-parallel to each other along the *c* direction, suggesting that those spins are coupled by an AFM *J*_6_ interaction (see Fig. 5a). The proposed scenario would then be the following: in the HT phase, the five Mn-Mn nearest neighbor interactions (*J*_1_ to *J*_5_) stabilize a complex spiral arrangement of the Mn spins. Note that, according to literature, *J*_5_ is AFM and likely the strongest interaction at play. Along *J*_4_ bonds, Mn^4+^ and Mn^3+^ are also anti-parallel to each other, suggesting that *J*_3_ is eventually the most frustrated coupling. Indeed, the effects of the different *J*_3_ on the magnetic energy cancel each other for a *Pbam* space group. It results that *J*_3_ only acts through weak symmetry breaking, and thus its global effect remains very small independently to its absolute value. The easy axis anisotropy of the Dy^3+^ spins competes with this underlying spiral order and maintains the Dy^3+^ spins along *b*. To take advantage of *J*_6_, however, the Dy^3+^ spins adopt this peculiar ↑↑↓↓ configuration. This is indeed a favorable compromise since for the sites where Mn^3+^ is along *b*, the *J*_6_ exchange energy is fully satisfied, while for the sites where Mn^3+^ is along *a*, Dy^3+^ and Mn^3+^ spins are perpendicular, so that the *J*_6_ exchange energy is zero.

Mean field calculations are then carried out using the SpinWave software^24^ to determine the magnetic structure at a certain temperature by means of energy minimization. The magnetic interactions at play are the *J*_1,…,5_ exchange couplings and the Mn^3+^-Dy^3+^ Ising coupling *J*_6_. By studying rigorously over a wide range of values, it was found that a parameter set corresponding to *J*_1_ = −1.5 meV, *J*_2_ = 1.5 meV, *J*_3_ = 0 meV, *J*_4_ = 1.75 meV, *J*_5_ = 3.5 meV and *J*_6_ = 0.03 meV could successfully yield a spiral magnetic structure similar to what is observed at 15 K with a propagation vector (1/2, 0, 1/4) (*J*_3_ cannot be evaluated due to cancellation effects by symmetry and has thus be assigned to 0). This analysis leads to the following relation for the magnitude of the exchange parameters: $\parallel {J}_{5}\parallel \ge \parallel {J}_{4}\parallel \gg \parallel {J}_{3}\parallel$, which is in line with previous analysis^25,26^.

Although small, the inclusion of *J*_6_ is essential to stabilize the structure similar to the 15 K one. We propose that since the ordered Dy^3+^ moment increases with decreasing temperature, the competition turns in favor of the *J*_6_ exchange energy. It finally overcomes *J*_1,2_, destabilizes the spiral order, and results in a structure where all Mn^3+^ and Dy^3+^ spins are anti-parallel. In this scenario, the magnetic phase transition is thus a manifestation of the competition among the exchange interactions and the anisotropy energy.

### Spin dynamics

To further investigate this scenario, inelastic neutron scattering measurements have been carried out both in the HT and LT phases.

**Figure 6:**
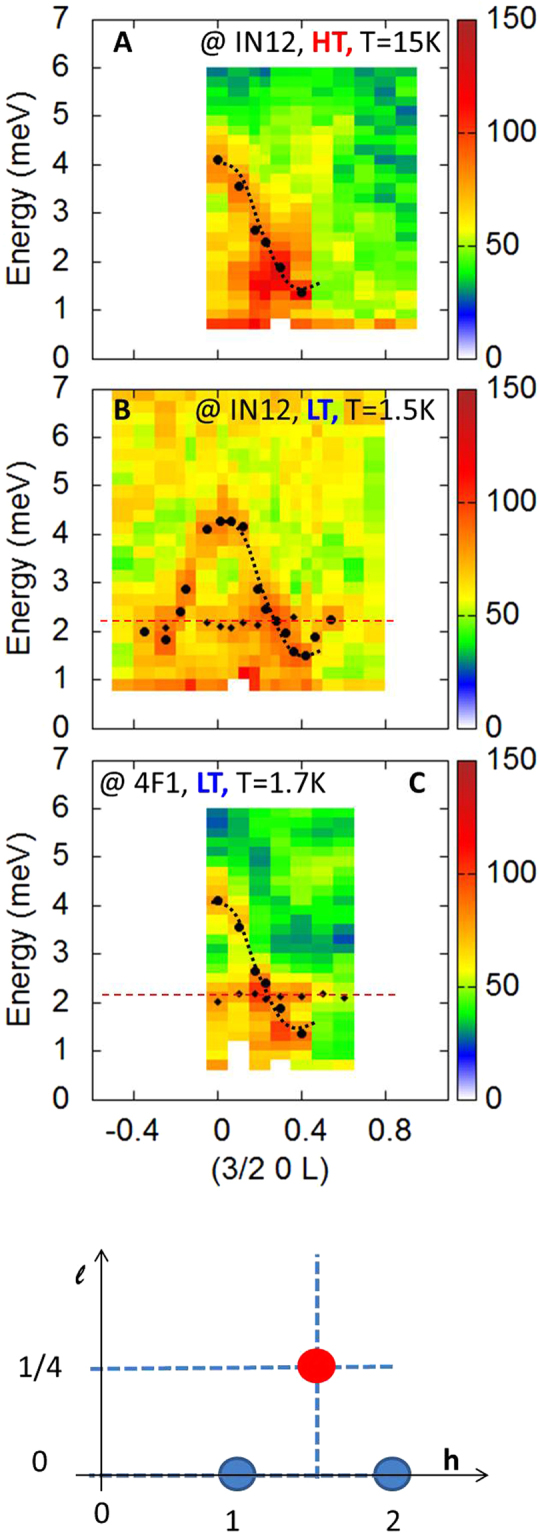
Inelastic neutron scattering measurements along c*. (**A**,**B**) Are cuts taken at 15 and 1.5 K respectively along $(3/2,0,\ell )$. (**C**) Shows the same data taken at the 4F1 spectrometer in a wider *Q* range. In (**B**,**C**), the diamonds show the fitted position of the nearly flat mode at Ω = 2 meV and the circles show the spin wave dispersion also fitted from the data. In these fits, we assumed a Gaussian profile. The dotted lines are guides to the eyes. The sketch depicts the (a*, c*) reciprocal plane along with the directions of the different cuts.

**Figure 7:**
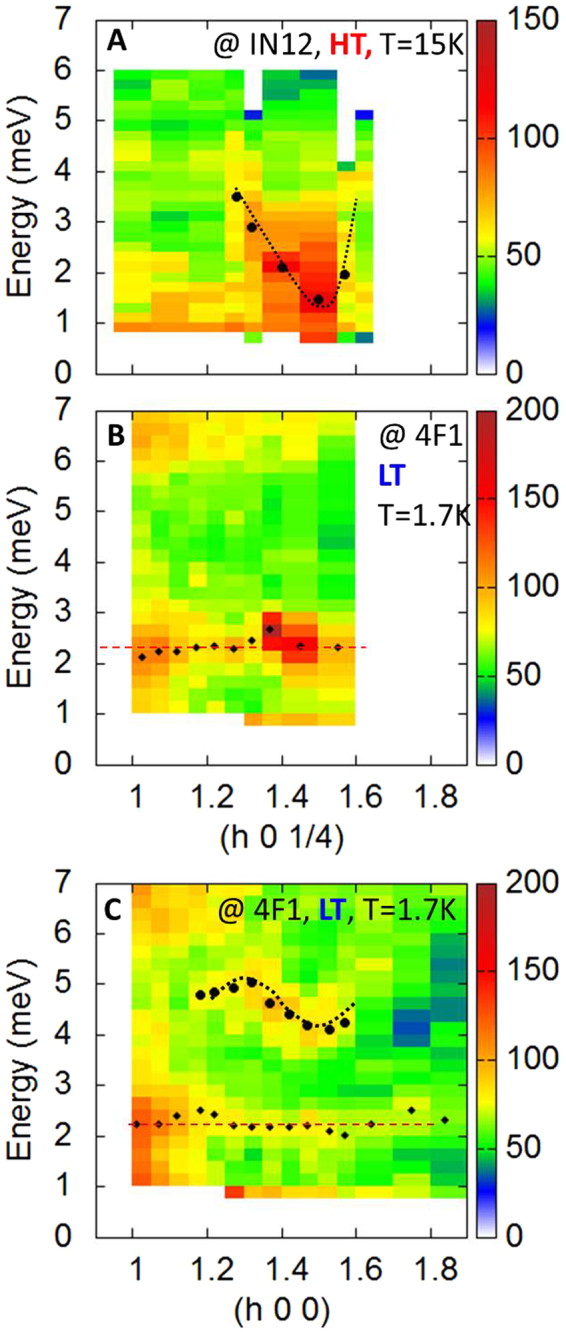
Inelastic neutron scattering measurements along a*. (**A**,**B**) Are cuts as a function of energy transfer and Q along (*h*, 0, 1/4) taken at 15 and 1.5 K respectively. (**C**) Shows a cut along (*h*, 0, 0) taken at low temperature. The diamonds highlight the flat mode at about Ω = 2 meV and the circles show the fitted spin wave dispersion. The dotted lines are guides to the eyes.

In standard magnets, an acoustic spin wave branch stems from the magnetic Bragg peak located at *Q*_Bragg_. This branch corresponds to the long wavelength precession of the spins around the average local magnetization. With this picture in mind, the vicinity of the HT phase Bragg peak, *Q*_Bragg_ = (3/2, 0, 1/4), has been mapped out as a function of energy transfer *ω* and *Q* wave-vector. Because of the small size (~mm^3^) of the sample, successful executions of the experiments were extremely challenging. All the measurements were thus repeated on different spectrometers (see Methods) to verify the genuineness of the data. Figures 6A and 7A display the neutron intensity measured at 15 K with *Q* along $(3/2,0,\ell )$, i.e. along c* and (*h*, 0, 1/4), i.e. along a* respectively (raw data). The directions of these two cuts in reciprocal space are depicted by dotted lines on the sketch presented in Fig. 6. Despite the weakness of the signal, different features can be noticed, which definitely resemble the spin wave response identified in the alike materials YMn_2_O_5_^25^ and TbMn_2_O_5_^26^. The data show indeed a feature (black dotted line) stemming from ${\rm{(3}}/2,0,\ell \approx {\rm{0.35)}}$ and dispersing upwards both along $\ell$ and *h*. We note that this is a bit off from the Bragg position, expected at $\ell \approx 1/4$. Furthermore, it is characterized by a gap Δ ≈ 1.5 meV. The branch also reaches a maximum around *ħω* = 4 meV at $\ell$ = 0, a behaviour again similar to that observed in YMn_2_O_5_^25^ and TbMn_2_O_5_^26^.

Figure 6B,C show the same data taken along c* in the LT phase at 2 K. Note that the wider $\ell$ range probed in C allows to demonstrate that the dispersion is symmetric with respect to $\ell =0$, as it should be for a spin wave. Surprisingly, those maps look pretty similar to the one at 15 K. This is an intriguing property, since the minimum of the dispersion, at this temperature, is expected at (3/2, 0, 0), i.e. the Bragg peak of the LT phase, and not close to (3/2, 0, 1/4). Furthermore, the data provide evidence for an additional nearly flat mode around *ħ*Ω = 2 meV. Along (*h*, 0, 1/4), see Fig. 7B, the acoustic spin wave is hard to detect, yet the intensity of the flat mode is strongly enhanced close to (3/2, 0, 1/4), as if the acoustic spin wave had collapsed on it. Along (*h*, 0, 0), however, the spin wave branch is again clearly visible in Fig. 7C, and corresponds to the upwards dispersion of the mode detected at 4 meV along c* (Fig. 6A–C) for $\ell =0$.

Such situations, where the acoustic spin wave branch does not stem from the Bragg peak of the magnetic structure, usually appear when exchange and anisotropy compete with each other, giving rise to “roton-like” minima in the dispersion^27^. Those minima appear at the *Q*_exch_ positions favored by exchange, yet the actual ground state is characterized by Bragg peaks different from *Q*_exch_ because of anisotropy. This is the case for instance in metallic Dysprosium, where a strong anisotropy destabilizes the high temperature helicoidal phase to the benefit of a low temperature ferromagnetic one^27^. A large anisotropy spin gap opens at low temperature, yet the spin wave branch continues to have minima at Q positions reminiscent of the helicoidal phase.

The inelastic neutron scattering experiments of Figs 6 and 7 integrate naturally in the scenario discussed above to account for the HT - LT transition. Indeed, the degeneracy of the ground doublet is lifted by the molecular field due to the Mn^3+^ spins, leading to a natural interpretation of the flat mode observed at Ω = 2 meV (see Fig. 7): the latter simply identifies with the transition between the split |↑〉 and |↓〉 states. At the mean field level, the splitting Ω and the Dy^3+^ magnetization *m* could be written as:

$${\rm{\Omega }}=z{J}_{6}\langle {m}_{{\rm{Mn}}}\rangle 2\frac{\mu }{{g}_{J}}$$

$$m=\mu \,tanh\,\frac{{\rm{\Omega }}}{2{k}_{{\rm{B}}}T}$$

where *g* = 2 is the 3*d* Landé factors, *z* = 2 is the number of Mn^3+^ neighbors of a given Dy^3+^ spin, and 〈*m*_Mn_〉 ≈ 3 *μ*_B_ is the average Mn^3+^ magnetic moment. From diffraction at 2 K, see Table 1, we measure *μ* ≈ 7.47 *μ*_B_ against 8.5 *μ*_B_ according to the theory; at 15 K, Table 2 gives *m* = 3.99 *μ*_B_ against *m* = 4.8 *μ*_B_ using Ω ≈ 2 meV, hence a good agreement. Finally, using Ω ≈ 2 meV, one gets *J*_6_ ≈ 0.06 meV. In the same spirit, we expect a modification of the Mn^4+^-Mn^3+^ spin dynamics, i.e. a modification of the spin wave spectra. Actually, the exchange *J*_6_ should, in principle, hybridize the crystal field and spin wave responses, as for instance proposed in other materials like ErMnO_3_^6^ or NdFe_3_(BO_3_)_4_^3^. This hybridization may be the physical origin of the peculiar spectrum observed in Fig. 7B, yet further theoretical studies are necessary to investigate this phenomenon.

## Summary and Conclusion

The magnetic structure and the spin dynamics in the multiferroic oxide DyMn_2_O_5_ have been investigated by means of neutron diffraction and inelastic scattering. A first order magnetic phase transition is reported at 8 K. It separates a high temperature magnetic structure spiraling around *c* direction with a propagation vector $(1/2,0,\ell \approx 1/4)$ and a low temperature antiferromagnetic configuration where spins are ferromagnetically stacked along *c* with a propagation vector (1/2, 0, 0). Although the high temperature phase persists to exist down to 2 K, the magnetic Bragg reflections become extremely weaker compared to the actual low temperature phase ones. Surprisingly, the nature of spin excitations is practically identical above and below the transition temperature along c*. Based on both kinds of experiments, we propose that the physics at play is presumably associated to the competition between exchange interactions and the single ion anisotropy of the Dy^3+^ ion. More generally, these findings demonstrate that the magnetic structure, hence the ferroelectricity, by virtue of the magneto-electric coupling, strongly depends on the anisotropy of the rare earth. The 3*d*-4*f* coupling thus appears as one of the key ingredients in the physics of multiferroics.

## Methods Summary

Single crystals of DyMn_2_O_5_ were synthesized following the method described in ref.^28^. Single crystal neutron diffraction experiments were conducted on the D23 diffractometer, a CEA-CRG instrument installed at the ILL (Grenoble, France). The instrument was operated in a four circles geometry with an incident wave length of 1.280(1) Å. The sample (2.5 × 1.7 × 1 mm^3^) was attached to the cold finger of a displex refrigerator. Its crystalline quality was checked using the OrientExpress instrument installed at ILL.

Inelastic neutron scattering (INS) measurements were performed at the 4F and IN12 cold triple axes spectrometers installed at LLB and ILL respectively, on the very same crystal used for the diffraction measurements. The final wave vectors used were *k*_*f*_ = 1.97 Å^−1^ and 1.8 Å^−1^ for 4F and IN12 respectively. To eliminate the higher order reflections, proper filtering was used. The sample was mounted to have access to $Q=(h,0,\ell )$ scattering wave-vectors and attached to the cold finger of an orange cryostat that can cool down to 1.7 K. Because of the small size (~*mm* ^3^) of the sample, INS experiments were extremely challenging. They were thus repeated on 4F and IN12 to verify the genuineness of the data. We then pursued the idea that features being common to the two sets of experiments should be retained only. It could be noticed, fortunately, that the spectra are close to each other, giving good confidence that the features, yet weak, are real. Furthermore, in the present manuscript, we chose to show the raw data; for instance, no background has been subtracted. To identify the signal, we compared the data with the spectra published for similar materials, YMn_2_O_5_ and TbMn_2_O_5_ in refs^25^ and^26^ respectively. Indeed, we observe features clearly reminiscent of the spin wave branches reported in these two compounds. The spin wave energies were determined based on a simple fit assuming a standard Gaussian profile on top of a constant background. A flat feature at about 6.5 meV may also correspond to a signal (see 7B and C). Actuallly, owing to the experimental set up, this energy region corresponds to small scattering angles, hence a region where the contamination due to the incident beam may become significant especially when considering long counting times. The feature at 6.5 meV is likely due to this effect, and thus not relevant.
